# Closure of the initial surgical wound resulting in successful treatment of a pediatric case of Fournier's gangrene: A case report

**DOI:** 10.1016/j.eucr.2024.102860

**Published:** 2024-10-02

**Authors:** Kohei Mori, Yutaka Shiono, Soichiro Shimura, Shuhei Hirano, Dai Koguchi, Masaomi Ikeda, Hideyasu Tsumura, Daisuke Ishii, Kazumasa Matsumoto

**Affiliations:** Department of Urology, Kitasato University School of Medicine, Japan

**Keywords:** Fournier's gangrene, Pediatric case

## Abstract

Fournier's gangrene is a severe type of necrotizing fasciitis that affects the perineal and genital regions. Because of its rapid progression, Fournier's gangrene is associated with high mortality and morbidity rates. Surgical treatment of Fournier's gangrene requires leaving the wound open and performing multiple debridement procedures. We report a case of Fournier's gangrene caused by *Streptococcus anginosus* in a 9-year-old boy with severe autism. Because of the patient's condition, surgical treatment included thorough debridement and closure of the initial wound under general anesthesia. This case was successfully treated and the patient was discharged without infection recurrence.

## 1 Introduction

Fournier's gangrene (FG) is a type of rapidly progressing necrotizing fasciitis that primarily affects the perineal, genital, and perianal regions. Described by Alfred Fournier in 1883, FG is associated with a high mortality rate because of its aggressive nature. FG commonly occurs in men 50–60 years of age and has an incidence of approximately 0.4 cases per 100,000 adults.^1^ However, FG can affect individuals of all ages.

Pediatric cases of FG are rare. Necrotizing fasciitis in various anatomical regions, including the perineal area, have been reported, with an incidence rate of 0.08 cases per 100,000 children.^2^ Etiological factors associated with pediatric cases of FG include omphalitis, strangulated hernia, premature birth, diaper rash, circumcision, and perineal skin abscesses.^3^, ^4^, ^5^

FG treatment includes extensive debridement of infected tissue and the administration of intravenous broad-spectrum antibiotics. Additionally, surgical treatment of FG requires leaving the wound open and performing multiple debridement procedures. However, maintaining an open wound is difficult in pediatric cases. We report the case of a 9-year-old boy with a rare case of FG that was successfully treated with surgical closure of the initial wound.

## 2 Case presentation

**Fig. 1:**
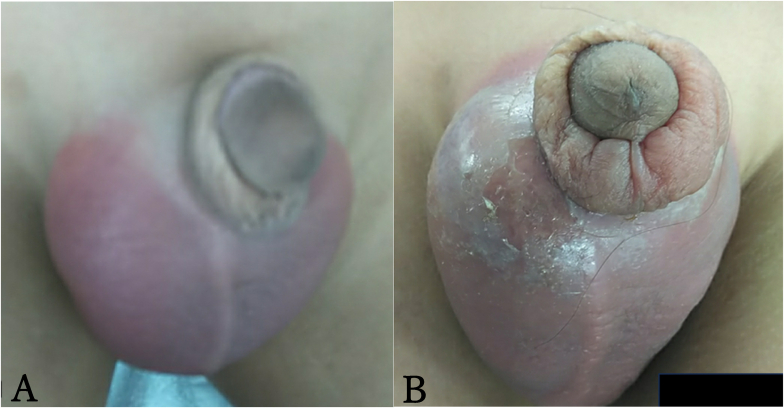
Erythema and swelling in the examination of the scrotum (A) The scrotum at the first visit. (B) The scrotum after the 5 days from the first visit. Erythema and swelling were worse mainly in the right scrotum.

**Fig. 2:**
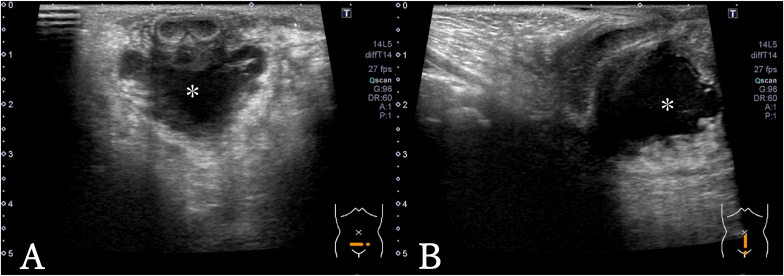
US examination of the scrotum (A) The transverse image. The low echoic lesion was observed on ventral side of urethra (∗). (B) The sagittal image. The low echoic lesion was observed on ventral side of urethra (∗).

A 9-year-old boy with severe autism presented to our emergency department with acute scrotal erythema (Fig. 1). An ultrasound examination of the scrotum revealed normal blood flow in the testicles; therefore, testicular torsion was considered unlikely. We suspected epididymis at that time and discharged the patient with treatment comprising oral antibiotics. Five days after the initial visit, the patient returned with worsening scrotal erythema, swelling, and fever (Fig. 1). Scrotal ultrasonography revealed a hypoechoic area (30 mm × 40 mm) in the right medial scrotum that was not observed during the initial examination (Fig. 2).

Laboratory tests showed a complete blood count of 29,200 leukocytes/mm^3^, 85 % segmented neutrophils, and increased acute-phase reactant levels (C-reactive protein level of 17.8 mg/dL). Severe abscess formation in the scrotum was suspected. After FG was diagnosed, surgical drainage was performed and new antibiotic treatment was administered.

**Fig. 3:**
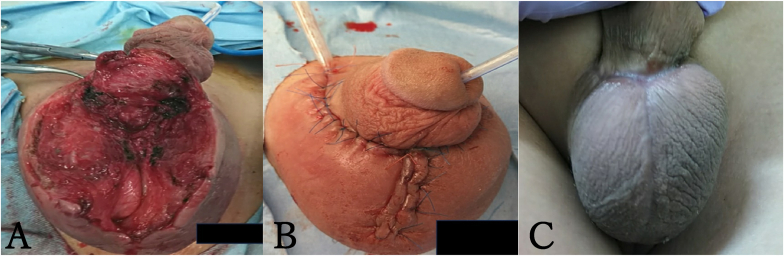
Intraoperative and postoperative wound picture. (A) Extensive scrotal subcutaneous debridement and skin excision. (B) Primary closure of the wound with drainage tube. (C) Surgical wound appearance 60 days post-operation.

A midline incision of the scrotum revealed pus. Extensive debridement of the necrotic tissue was performed under general anesthesia (Fig. 3). The scrotal secretion culture results revealed *Actinomyces species* and *Streptococcus anginosus*. Postoperative wounds associated with FG are managed openly. However, because our patient had severe autism, wound management was difficult; therefore, we decided to close the wound with drainage tube (Fig. 3).

During the first 3 days postoperatively, the patient was sedated using dexmedetomidine to ensure the stable administration of antibiotics (ampicillin/sulbactam plus clindamycin) and maintain the wound. The sutures were removed on postoperative day 10, and the patient was discharged on postoperative day 14.

At 60 days postoperatively, infection recurrence was not observed and the wound remained closed (Fig. 3). At 1 year postoperatively, clinical problems were not observed.

## 3 Discussion

FG is a severe and quickly progressing type of necrotizing fasciitis caused by a polymicrobial infection that affects the perineal area and genitalia.^6^ Mortality rates associated with FG range from 11 % to 45 %.^7^ The necrotizing process is typically initiated by an infection in the anorectum, urogenital tract, or genitalia skin 8. Bacteria produce enzymes, such as collagenase and hyaluronidase, that infiltrate the fascial planes, thus causing vascular thrombosis and subsequent gangrene of the overlying skin 9. These bacteria continue to multiply in the necrotic tissues.

Pediatric cases of FG are rare. Necrotizing fasciitis in various anatomical regions, including the perineal area, have been documented, with an incidence rate of 0.08 cases per 100,000 children.^2^ Scrotal abscesses can be caused by appendicitis, duodenal perforation, colonic perforation, Cowper's duct cyst infection, and pancreatitis.^10^, ^11^, ^12^, ^13^, ^14^ However, our case had no identifiable cause.

The primary therapeutic approach for FG comprises the use of broad-spectrum intravenous antibiotics and extensive surgical debridement. Multiple debridement procedures may be required to remove all necrotic tissue, reduce the bacterial load, and control infection. However, maintaining an open wound can be particularly challenging when the patient is a child, as demonstrated by our case. Therefore, primary wound closure and thorough debridement of the necrotic tissue during the initial surgery should be considered for pediatric FG cases. To the best of our knowledge, this is the first reported pediatric FG case involving closure of the initial wound.

This case highlights the importance of an early diagnosis and aggressive treatment for pediatric cases of FG. Unlike adult patients, pediatric patients often present unique challenges, such as difficult open wound maintenance and the presence of different etiological factors. Further research is necessary to obtain a better understanding of the long-term outcomes of pediatric FG and develop standardized treatment protocols that can be applied across healthcare settings. Additionally, this case emphasizes the need for increased clinical vigilance of FG, especially when it is not initially suspected and particularly for pediatric patients with atypical presentations.

## 4 Conclusion

FG caused by *Streptococcus anginosus* was effectively treated with extensive debridement and primary wound closure. Because of the difficulty associated with managing the open wound of pediatric patients, primary wound closure may be an appropriate treatment option. This case highlights a new surgical management option for pediatric FG and the potential to use alternative approaches for similar cases.

## Informed consent

The patient's father provided informed consent prior to study participation.

## Funding

This work was produced without outside funding contributions.

## Data availability

Not applicable.

## CRediT authorship contribution statement

**Kohei Mori:** Writing – original draft, Data curation, Conceptualization. **Yutaka Shiono:** Data curation, Conceptualization. **Soichiro Shimura:** Data curation, Conceptualization. **Shuhei Hirano:** Conceptualization. **Dai Koguchi:** Conceptualization. **Masaomi Ikeda:** Conceptualization. **Hideyasu Tsumura:** Conceptualization. **Daisuke Ishii:** Conceptualization. **Kazumasa Matsumoto:** Supervision.

## Declaration of competing interest

The authors have no conflicts of interest relevant to this article to disclose.
